# Определение референсных интервалов пролактина для различных возрастных групп

**DOI:** 10.14341/probl13095

**Published:** 2023-06-30

**Authors:** Г. С. Колесникова, Н. М. Малышева, З. Т. Зураева, Л. В. Никанкина, Г. А. Мельниченко

**Affiliations:** Национальный медицинский исследовательский центр эндокринологии; Национальный медицинский исследовательский центр эндокринологии; Национальный медицинский исследовательский центр эндокринологии; Национальный медицинский исследовательский центр эндокринологии; Национальный медицинский исследовательский центр эндокринологии

**Keywords:** пролактин, референсные интервалы, VITROS

## Abstract

**ОБОСНОВАНИЕ:**

ОБОСНОВАНИЕ. В современных диагностических лабораториях представлен широкий спектр тест-систем и автоматизированных платформ для определения уровней различных биохимических показателей, в частности, пролактина. Каждый из производителей тест-систем предлагает свой вариант метода, что затрудняет стандартизацию. На практике часто возникает проблема расхождения результатов лабораторных исследований и референсных интервалов, предоставляемых разработчиками, с клинической картиной пациентов. Это определяет необходимость выработки метод-специфичных референсных интервалов для конкретной популяции. Сложность интерпретации базального уровня пролактина связана не только с повышением уровня гормона, вызванным стрессом или чрезмерными физическими нагрузками, но и со значительной вариабельностью показателей у одного и того же пациента даже при соблюдении всех рекомендаций по забору крови.

**ЦЕЛЬ:**

ЦЕЛЬ. Установить референсные интервалы концентраций пролактина у женщин, мужчин и детей различных возрастных групп для автоматизированной системы Vitros и сравнить полученные результаты с референсными интервалами, предоставленными производителем тест-системы.

**МАТЕРИАЛЫ И МЕТОДЫ:**

МАТЕРИАЛЫ И МЕТОДЫ. В исследование включено 879 образцов сыворотки крови условно здоровых людей, проходивших обследование в ФГБУ «НМИЦ эндокринологии» Минздрава России. Для проведения измерений использовали автоматический хемилюминесцентный анализатор VITROS ECi 3600 (Ortho-Clinical Diagnostics, Великобритания).

**РЕЗУЛЬТАТЫ:**

РЕЗУЛЬТАТЫ. Для взрослых мужчин и женщин референсные интервалы составили 66–436 и 94–500 мЕд/л соответственно. При сравнении результатов определения пролактина референсной группы репродуктивного возраста с «ожидаемыми значениями» производителя тест-системы VITROS ECi 3600 был обнаружен систематический сдвиг в сторону увеличения верхней границы референсных значений.

**ЗАКЛЮЧЕНИЕ:**

ЗАКЛЮЧЕНИЕ. Полученные референсные интервалы могут быть рекомендованы для использования при определении уровня пролактина в российской популяции тест-системами VITROS ECi 3600.

## Abstract (en)

**BACKGROUND:**

BACKGROUND: Clinical diagnostic laboratories (CDL) have at their disposal various automated systems for the measurement of biochemical parameters and markers such as prolactin. Each of the test systems manufactures offers its own alternate design of the method, which makes standardization difficult. In endocrinological practice, the problem of result discrepancies often arises. In hormonal assays, the clinical picture of patients sometimes does not correspond to the reference values provided by the manufacturers, which determines the need to develop the method-specific reference values for a specific population. The difficulty in interpreting basal prolactin levels is not only due to a transient increase in the hormone levels caused by stress or excessive physical exertion, but also due to a significant variability of indicators in the same patient, even if all recommendations for blood sampling are followed.

**AIM:**

AIM: The aim of the study to determine the reference values in serum samples of women, men and children of different age groups for the VITROS Immunodiagnostic Systems and compare the results with the “expected prolactin levels” recommended by the method manufacturer

**MATERIALS AND METHODS:**

MATERIALS AND METHODS: The monocentric study included 879 serum samples of apparently healthy subjects who were admitted to the Endocrinology Research Center. Measurements were performed using the VITROS ECi 3600 automatic chemiluminescence analyzer (Ortho-Clinical Diagnostics, Great Britain).

**RESULTS:**

RESULTS: For adult men and women reference values were 66–436 and 94–500 mIU/l respectively. When comparing the results of prolactin determination in women and men of the studied cohort with the “expected values” of the VITROS ECi 3600 manufacturer a systematic shift towards an increase in lower and upper limits of the reference values was found in both ­cohorts.

**CONCLUSION:**

CONCLUSION: The obtained reference values of prolactin can be use in the determination of prolactin in the Russian population for VITROS ECi 3600 Immunodiagnostic Systems.

## ОБОСНОВАНИЕ

Пролактин — полипептидный гормон, секретируемый лактотрофными клетками передней доли гипофиза. Пролактин обладает широким спектром биологического действия в организме человека, играет важную роль в становлении репродуктивной функции как мужчин, так и женщин, а также в обеспечении запуска и поддержания процесса лактации и нормального функционирования желтого тела. Под контролем пролактина находятся и другие функции организма, патологические изменения секреции данного гормона как в сторону повышения, так и в сторону снижения приводят к угнетению сперматогенеза и снижению секреции тестостерона у мужчин и нарушению менструального цикла у женщин, что приводит к гипогонадизму и бесплодию. Кроме того, пролактин оказывает непосредственное влияние на состояние иммунной системы (способствует пролиферации Т- и В-лимфоцитов), водно-электролитный (осморегуляция), углеводный и жировой обмен (повышение активности глюкокиназы и гликогенфосфорилазы в гепатоцитах, в частности, участвует в регуляции чувствительности к инсулину), определяет многие поведенческие и психологические реакции (устойчивость к стрессам, забота о потомстве). Таким образом, под контролем пролактина находятся практически все звенья метаболизма в организме человека [1–5].

Снижение секреции пролактина — достаточно редкое явление, в основном к этому может привести хирургическое вмешательство (удаление опухоли гипофиза). Причины, приводящие к повышению секреции пролактина, более многочисленны:

Таким образом, совершенно ясно, что наличие метод-специфичных референсных интервалов при определении концентрации пролактина в сыворотке крови пациентов имеет чрезвычайно важное значение. Правильность интерпретации результатов исследования определяется точностью измерения концентрации пролактина и зависит от используемого метода анализа.

В распоряжение клинико-диагностических лабораторий предоставлен широкий выбор тест-систем для определения всевозможных биохимических показателей и маркеров, в частности, пролактина. Любой из существующих методов иммуноанализа основан на принципе взаимодействия антиген-антитело, однако требует индивидуальных технологических разработок для каждого аналита, включая получение моноклональных антител с высокой специфичностью для повышения его чувствительности. Каждый производитель конструирует свой вариант метода, что затрудняет стандартизацию. В эндокринологической практике часто возникает проблема расхождения результатов гормональных исследований и референcных значений, предоставляемых разработчиками, с клинической картиной пациентов, что определяет необходимость выработки метод-специфичных референcных интервалов для конкретной популяции. Сложность интерпретации показателей базального уровня пролактина обусловлена не только транзиторным повышением концентрации гормона при стрессах или чрезмерных физических нагрузках, но и существенной вариабельностью показателей у одного и того же больного

## ЦЕЛЬ ИССЛЕДОВАНИЯ

Установить референсные интервалы концентраций пролактина у женщин, мужчин и детей различных возрастных групп российской популяции для автоматизированной системы Vitros и сравнить полученные результаты с референсными интервалами, предоставленными производителем тест-системы.

## МАТЕРИАЛЫ И МЕТОДЫ

## Место и время проведения исследования

Место проведения. ФГБУ «НМИЦ эндокринологии» Минздрава России.

Время исследования. Период с июля 2019 г. по июль 2020 г.

## Изучаемые популяции (одна или несколько)

В исследование были включены образцы сывороток условно здоровых субъектов с нормальными параметрами клинической биохимии, гематологического и гормонального анализа крови, то есть находившиеся в стадии клинической ремиссии. После анализа историй болезни из исследования были исключены пациенты, имеющие следующие заболевания в анамнезе:

В число отобранных вошли 879 пациентов, включая детей 5–18 лет и взрослых 19–85 лет.

## Способ формирования выборки из изучаемой популяции (или нескольких выборок из нескольких изучаемых популяций)

Сплошной.

## Дизайн исследования

Одномоментное моноцентровое исследование.

## Методы

Процедура сбора крови

Образцы крови были взяты из локтевой вены между 8.00 и 10.00 утра, у женщин в фолликулиновую фазу цикла. Сыворотку получали путем центрифугирования при температуре 4ºС и 3000 об/мин. Образцы сыворотки хранили при -20ºС в аликвотах до измерения в них содержания пролактина.

Автоматизированная система

Для проведения измерений использовали автоматический хемилюминесцентный анализатор VITROS ECi 3600 (Ortho-Clinical Diagnostics, Великобритания). Измерение проводили по стандартной методике разработчика тест-системы с помощью теста для количественного определения пролактина в сыворотке и плазме крови человека VITROS Immunodiagnostic Products Prolactin REF 184 9793 [6].

## Статистический анализ

Статистическую обработку полученных данных осуществляли с помощью компьютерной программы STATISTICA 7 (StatSoft, IncUSA) непараметрическим методом. Данные представлены в виде медианы, интерпроцентильного размаха между 2,5 и 97,5% перцентилями. Референсные интервалы рассчитаны согласно рекомендациям Института клинических и лабораторных стандартов (CLSI) для небольших размеров выборки (менее n=120) с использованием программного обеспечения MedCalc версии 19.6.4 [7]. Графики и линейную регрессию выполняли с помощью GraphPad Prism Version 9.0.2.

## Этическая экспертиза

Исследование было одобрено этическим комитетом ФГБУ «НМИЦ эндокринологии» Минздрава России 8 мая 2019 г., протокол №8.

## РЕЗУЛЬТАТЫ

Результаты определения концентрации пролактина у девочек 5–18 лет по годам представлены на рис. 1 и в табл. 1. На рисунке хорошо видно, что по мере взросления и, следовательно, полового созревания уровень пролактина постепенно увеличивается. В группе женщин медианы были рассчитаны для каждых 10 лет (рис. 2, табл. 2). С возрастом, то есть «затуханием» репродуктивной функции, наблюдается снижение уровня пролактина. По полученным данным были сформированы логические возрастные интервалы, отражающие изменение уровня пролактина у девочек и женщин на протяжении жизни (табл. 3). Девочки были разделены на следующие подгруппы: в возрасте 5–8 лет — до полового созревания, 9–13 лет — начало пубертата, 14–18 лет — становление нормальной репродуктивной функции. Для женщин было выделено два возрастных интервала: репродуктивный возраст (до 49 лет) и постменопауза (более 50 лет).

**Figure:**
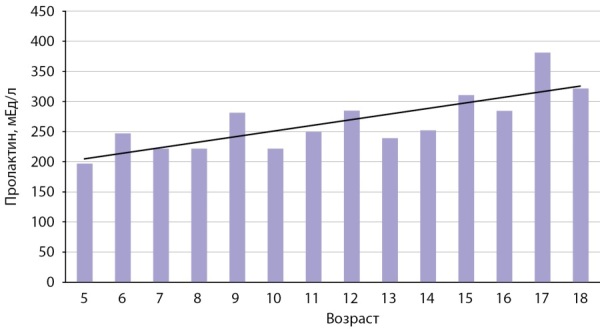
Рисунок 1. Показатели медиан пролактина (мЕд/л) у девочек и девушек в зависимости от возраста.Figure 1. Indicators of median prolactin (mU / l) in girls and girls, depending on age

**Table:** Таблица 1. Показатели пролактина, определенные на автоматическом анализаторе Vitros, у девочек и девушек, мальчиков и юношейTable 1. Prolactin values determined on the Vitros automatic analyzer in girls and girls, boys and boys

Возраст, лет	Девочки и девушки	Мальчики и юноши
n	Me [ 2,5; 97,5]	n	Me [ 2,5; 97,5]
5	18	198,6 [ 80,5; 470,9]	10	229,3 [ 121,6; 411,5]
6	19	247,1 [ 147,3; 468,2]	14	218,5 [ 133,5; 338,5]
7	19	222,1 [ 107,8; 465,1]	18	254,2 [ 107,0; 388,4]
8	20	221,0 [ 149,8; 435,7]	13	253,8 [ 118,9; 390,5]
9	19	281,7 [ 187,4; 467,5]	19	193,4 [ 94,3; 357,3]
10	12	221,1 [ 135,2; 360,3]	21	227,0 [ 107,4; 454,0]
11	18	249,7 [ 156,2; 502,6]	15	288,2 [ 138,9; 403,8]
12	19	284,2 [ 159,7; 453,2]	25	244,2 [ 150,2; 447,7]
13	13	240,4 [ 136,1; 450,4]	8	229,9 [ 131,5; 451,2]
14	16	253,2 [ 135,6; 482,2]	25	258,7 [ 127,5; 421,8]
15	14	308,9 [ 171,3; 479,8]	17	313,5 [ 139,9; 462,6]
16	14	284,3 [ 159,7; 469,5]	11	271,1 [ 178,1; 396,3]
17	13	381,1 [ 129,0; 539,5]	12	363,8 [ 254,0; 482,6]
18	21	322,0 [ 159,0; 538,3]	9	300,5 [ 136,9; 392,7]

**Figure:**
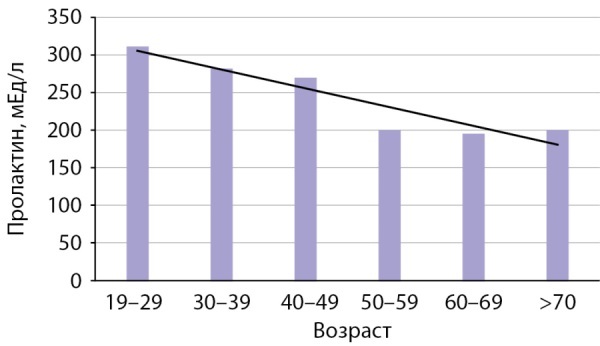
Рисунок 2. Показатели медиан пролактина (мЕд/л) у взрослых женщин в зависимости от возраста.Figure 2. Median prolactin values (mU/l) in adult women depending on age.

**Table:** Таблица 2. Показатели пролактина, определенные на автоматическом анализаторе Vitros, у женщин и мужчинTable 2. Prolactin values determined on the Vitros automatic analyzer in women and men

Возраст, лет	Женщины	Мужчины
n	Me [ 2,5; 97,5]	n	Me [ 2,5; 97,5]
19–29	46	309,6 [ 181,5; 493,4]	38	245,6 [ 125,0; 449,6]
30–39	43	282,1 [ 141,4; 477,7]	50	232,6 [ 145,7; 430,1]
40–49	31	269,8 [ 101,7; 493,9]	32	192,7 [ 114,7; 374,8]
50–59	25	199,4 [ 97,8; 329,4]	39	204,9 [ 112,3; 405,6]
60–69	35	195,0 [ 114,0; 394,0]	44	209,3 [ 103,1; 436,6]
Старше 70	11	200,9 [ 120,4; 359,8]	21	214,6 [ 115,8; 371,5]

**Table:** Таблица 3. Показатели пролактина, определенные на автоматическом анализаторе Vitros, у девочек и женщин в возрастных группахTable 3. Prolactin values determined on the Vitros automatic analyzer in girls and women in age groups

Возраст, лет	n	Me [ 2,5; 97,5]
5–8	76	221,6 [ 88,5; 445,0]
9–13	81	264,7 [ 130,4; 484,9]
14–18	78	308,9 [ 123,9; 515,4]
19–49	120	284,7 [ 125,5; 497,2]
Старше 50	83	198,9 [ 102,9; 374,6]

Pезультаты определения концентрации пролактина у мальчиков 5–18 лет представлены на рис. 3 и в табл. 1. Так же, как у девочек, наблюдается увеличение уровня пролактина к 15–18 годам до взрослого уровня. У мужчин старше 19 лет с возрастом наблюдается незначительное снижение уровня пролактина, менее заметное, чем у женщин (рис. 4, табл. 2).

**Figure:**
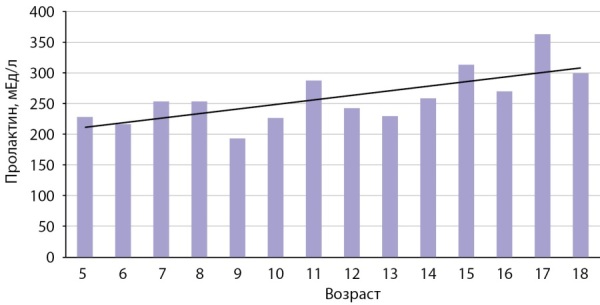
Рисунок 3. Показатели медиан пролактина (мЕд/л) у мальчиков и юношей в зависимости от возраста.Figure 3. Indicators of median prolactin (mU/l) in boys and young men depending on age

**Figure:**
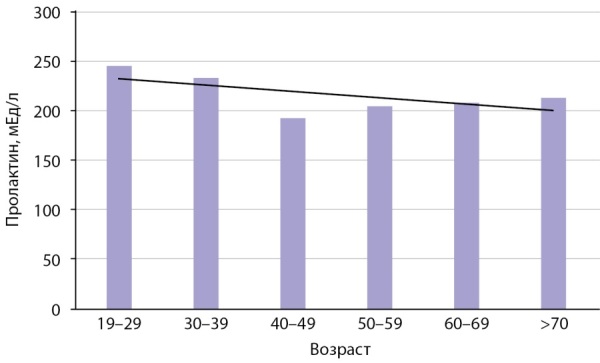
Рисунок 4. Показатели медиан пролактина у взрослых мужчин (мЕд/л) в зависимости от возраста.Figure 4. Median prolactin levels in adult men (mU/l) depending on age.

По полученным данным были сформированы логические возрастные интервалы, отражающие изменение уровня пролактина у мальчиков и мужчин на протяжении жизни (табл. 4). Мальчики были разделены на следующие подгруппы: в возрасте 5–9 лет — до полового созревания, 10–13 лет — начало пубертата, 14–18 лет — становление нормальной репродуктивной функции. Для мужчин было выделено также два возрастных интервала: 19–39 лет и старше 40 лет.

**Table:** Таблица 4. Показатели пролактина, определенные на автоматическом анализаторе Vitros, у мальчиков и мужчин в возрастных группахTable 4. Prolactin values determined on the Vitros automatic analyzer in boys and men in age groups

Возраст, лет	n	Me [ 2,5; 97,5]
5–9	74	229,5 [ 96,4; 421,5]
10–13	69	239,0 [ 117,0; 458,5]
14–18	74	293,9 [ 125,9; 476,9]
19–39	88	228,3 [ 113,0; 431,9]
Старше 40 лет	136	209,4 [ 99,2; 417,0]

При сравнении результатов определения концентрации пролактина у женщин и мужчин референсной группы с «ожидаемыми значениями» производителя VITROS ECi 3600 (64–395 мЕд/л для женщин и 78–380 мЕд/л для мужчин) был обнаружен систематический сдвиг в сторону увеличения как нижней, так и верхней границы референсных значений в обеих группах. Исходя из полученных данных, нами предложены следующие референсные интервалы при определении пролактина с помощью автоматизированной системы VITROS ECi 3600 (табл. 5).

**Table:** Таблица 5. Референсные интервалы пролактина (мЕд/л) в сыворотке крови, установленные с использованием автоматизированной системы VITROS ECi 3600, для женщин, мужчин и детей различных возрастных групп российской популяцииTable 5. Reference intervals of prolactin (mU/l) in blood serum, established using the automated system VITROS ECi 3600, for women, men and children of different age groups of the Russian population

Пол	Возраст, лет	n	РИ
Женский	5–8	76	90–450
9–13	81	130–490
14–18	78	125–520
19–49	120	110–500
Старше 50	71	110–400
Мужской	5–9	74	95–420
10–13	69	110–460
14–18	74	120–480
19–39	88	110–430
Старше 40	136	100–420

## ОБСУЖДЕНИЕ

Лабораторные исследования являются неотъемлемой частью процесса принятия клинических решений. Поэтому точность измерений и правильность интерпретации приобретают особое значение. Однако существуют значительные различия не только в референсных интервалах показателей пролактина у здоровых людей, предлагаемых различными производителями (табл. 6), но и в абсолютных значениях в одной и той же пробе (рис. 5), определенных разными методами [16]. Так, в некоторых образцах сыворотки (пациенты 6–9, рис. 5) может обнаруживаться высокомолекулярная форма пролактина — макропролактин, обладающий ограниченной биологической активностью in vivo, но сохраняющий иммунореактивность. Несмотря на то, что большинство предлагаемых для практического применения методов иммуноанализа основано на принципе взаимодействия антиген-антитело, все они различаются по участкам распознавания молекул, источникам получения антител, используют различные виды меченых соединений и т.д., в связи с чем имеют различную специфичность и чувствительность. Следовательно, в одном и том же образце сыворотки одни и те же показатели, полученные разными тест-системами, могут заметно отличаться по абсолютной величине. Эта разница отражается в референсных интервалах, разработанных производителем для каждой конкретной тест-системы иммуноанализа. Каждая лаборатория должна исследовать применимость ожидаемых значений к популяции своего региона и при необходимости определить собственный диапазон референсных значений.

**Table:** Таблица 6. Референсные интервалы пролактина (мЕд/л), разработанные производителем для каждой конкретной тест-системыTable 6. Prolactin reference intervals (mU/l) developed by the manufacturer for each specific test system

Мужчины	Женщины	Тест-система	Ссылка на источник литературы
44,7–227,9	83,1–432,4	Delfia	8
	106,5–447,3	Cobas 411	3
55,6–340,8		ELISA (US)	9
	178,9–757,5	Elecsys	10
73,4–413,2	110,3–565,1	Architect	11
58–419	63–561	Architect	12
42,6–340,8	63,9 -617,7	Centaur	13
42,6–340,8	63,9–681,6	Immulite2000	13
56,7–275, 1	74,1–388,5	Elecsys	14
Мальчики	Девочки		
68,1–392,2	68,1–392,2	CLIA (Beckman Coulter)	15

**Figure:**
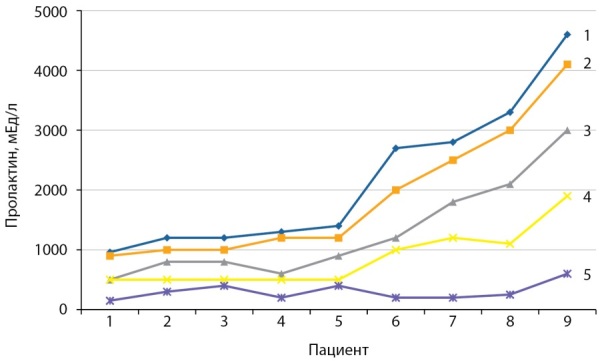
Рисунок 5. Определение уровня пролактина у 9 различных пациентов различными автоматизированными системами: 1 — Elecsys 411, 2 — Delfia, 3 — Architect 2000, 4 — Immulite 2000, 5 — гельфильтрация.Figure 5. Determination of the level of prolactin in 9 different patients by various automated systems: 1 - Elecsys 411, 2 - Delfia, 3 - Architect 2000, 4 - Immulite 2000, 5 - gel filtration.

Полученные нами данные демонстрируют изменение уровня пролактина в крови с возрастом и со становлением/угасанием репродуктивной функции. Выявлен широкий диапазон концентрации пролактина в сыворотке крови, при этом наблюдаются относительно небольшие возрастные изменения и незначительная гендерная разница в подростковом возрасте. Референсные интервалы, установленные в этом исследовании, позволяют повысить точность и правильность интерпретации результатов определения пролактина с помощью автоматического иммуноанализатора VITROS ECi 3600.

Исходя из полученных данных, необходимо подчеркнуть важность правильной интерпретации результатов определения пролактина при первичной диагностике, однако еще более важным является использование одного и того же метода при лечении и долгосрочном наблюдении пациента. Результаты, получаемые с помощью любого диагностического набора, могут быть интерпретированы только в контексте общей клинической картины. Окончательный диагноз определяется совокупностью клинических симптомов болезни и биохимических параметров пациента, опытом и знаниями лечащего врача.

## ЗАКЛЮЧЕНИЕ

Референсные значения пролактина, разработанные клинико-диагностической лабораторией ФГБУ «НМИЦ эндокринологии» Минздрава России для российской популяции, могут быть рекомендованы специалистам, которые сотрудничают с лабораториями, использующими автоматизированную систему VITROS ECi 3600 (Ortho-Clinical Diagnostics).

## ДОПОЛНИТЕЛЬНАЯ ИНФОРМАЦИЯ

Источники финансирования. Работа выполнена по инициативе авторов без привлечения финансирования.

Конфликт интересов. Авторы заявляют об отсутствии конфликта интересов.

Участие авторов. Колесникова Г.С. — концепция и дизайн исследования, статистическая обработка материала, написание и редактирование текста; Никанкина Л.В. — концепция и дизайн исследования, редактирование текста; Малышева Н.М. — сбор и обработка материала, написание текста; Зураева З.Т. — сбор и статистическая обработка материала; Мельниченко Г.А. — редактирование финального текста рукописи.

Все авторы одобрили финальную версию статьи перед публикацией, выразили согласие нести ответственность за все аспекты работы, подразумевающую надлежащее изучение и решение вопросов, связанных с точностью или добросовестностью любой части работы.
